# Co-infection of *Echinococcus equinus* and *Echinococcus canadensis* (G6/7) in a gray wolf in Turkey: First report and genetic variability of the isolates

**DOI:** 10.1016/j.ijppaw.2023.01.006

**Published:** 2023-01-18

**Authors:** Seyma Gunyakti Kilinc, Figen Celik, Harun Kaya Kesik, Muhammed Ahmed Selcuk, Haroon Ahmed, Sami Simsek

**Affiliations:** a Department of Parasitology, Faculty of Veterinary Medicine, Bingol University, Bingol, Turkey; b Department of Parasitology, Faculty of Veterinary Medicine, University of Firat, 23119, Elazig, Turkey; c Department of Parasitology, Faculty of Veterinary Medicine, Siirt University, Siirt, Turkey; d Department of Biosciences, COMSATS University Islamabad (CUI), Park Road, Chakh Shahzad, Islamabad, Pakistan

**Keywords:** *Echinococcus equinus*, *E. canadensis* (G6/7), Gray wolf, Haplotype, Turkey

## Abstract

Cystic echinococcosis (CE) is an important zoonotic diseases caused by larval form of *Echinococcus granulosus sensu lato*. The material of this study was the gray wolf (*Canis lupus*), which was found dead in the rural area of Bingol province of Turkey. The animal was brought to Veterinary Faculty for necropsy and many of adult *Echinococcus* spp. obtained. A total of 9 whole adult worms were morphologically examined under the microscope, gDNA was isolated from individual samples, a partial mt-CO1 gene fragment (875 bp) was amplified with PCR and sequenced. According to the phylogenetic analysis, six worms were characterized as *E. equinus*, while three were reported as *E. canadensis* (G6/7). It was found that the haplotypes of both species were similar to previously published haplotypes. This is the first report in which *E. equinus* and *E. canadensis* (G6/7) adult parasites were detected together in a gray wolf's intestine*.* The findings are important in that it draws attention to the importance of wild cycle in the spread of CE.

## Highlights

• Nine adult worms were collected from the intestine of a gray wolf.
• A partial mt-CO1 gene fragment (875 bp) was amplified and sequenced*.*
• Six worms were characterized as *E. equinus*, while three were *E. canadensis* (G6/7).
• This is the first report in which *E. equinus* and *E. canadensis* (G6/7) were detected together in a gray wolf.

## Graphical abstract

**Figure:**
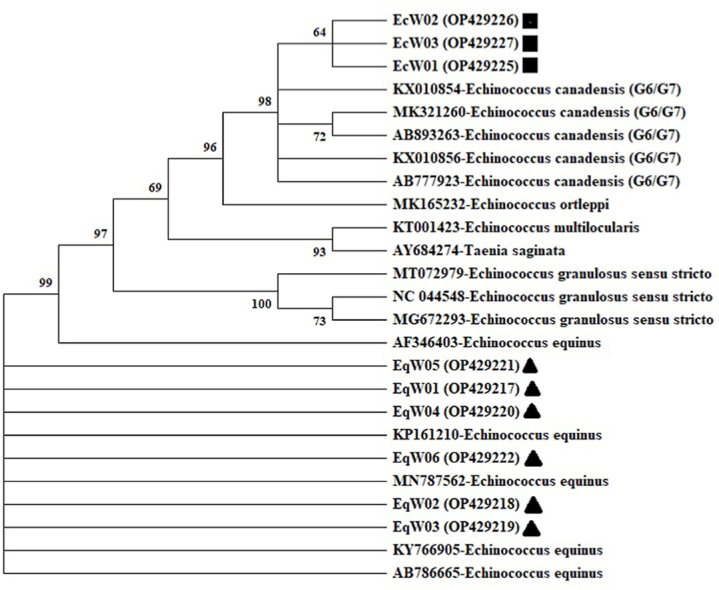

## 1 Introduction

*Echinococcus* species circulate among domestic and wild animal hosts that are occasionally transmitted to human and are known as the causative agent of cystic echinococcosis (CE). Although *Echinococcus* species affect wildlife, livestock animals and humans worldwide and limited data are avaliable regarding prevalence and genetic diversity of *Echinococcus* species in wildlife (Oksanen and Lavikainen, 2015). Due to a little data on population density and predation rates, it is impossible to determine the impact of specific host species on transmission (Romig et al., 2017). Although *E. granulosus sensu lato* appears to be a especially well-adapted species for sheep, its ancestral wildlife cycle includes intermediate hosts (mouflon, cervids, and wild boars) and definitive hosts (wolves and foxes) transmission (Romig et al., 2017). Molecular techniques have been highly useful in resolving classification confusion, mostly clarifying unique taxonomic implications and, more relevantly, the reliability of differential morphological features from a practical viewpoint (McManus, 2013). After 20 years of collecting epidemiological, biochemical, and geographic reports on *E. granulosus* isolates, as well as comprehensive phylogenetic analysis of nuclear and mitochondrial genes, restriction and conﬂicts of classification system within *E. granulosus* became obvious, necessity a genomic revision (Romig et al., 2015). Thus, E. granulosus s.l. is currently classified into E. granulosus sensu stricto (s.s.) (G1 and G3), Echinococcus felidis, Echinococcus equinus, Echinococcus ortleppi and Echinococcus canadensis (G6/G7, G8 and G10) (Vuitton et al., 2020). *E. granulosus s.l*. primarily circulates among dogs and different intermediate hosts, however, it does occasionally involve a predator-prey wildlife cycle that is unaffected by anthropogenic intervention (Tamarozzi et al., 2020)*.*

*E. granulosus s.s*., *E. equinus* and *E. ortleppi* have a domestic life cycle that includes mainly dogs and some livestocks (Carmena and Cardona, 2013). Whereas, *E. canadensis* (G6/7) can be transferred via both domestic and wildlife cycles (Nakao et al., 2013). Since *Echinococcus* species differ markedly in their host infectiousness, geographic prevalence, zoonotic condition, form and duration of development, pathogenicity, data from molecular epidemiology are precious for estimating the genotype/haplotype densities of the worm in an area as well as revealing transmission dynamics (McManus, 2013).

The gray wolves (*Canis lupus*) spread in an area of approximately 500,000 km^2^ in all regions of Turkey, mainly in the Central Anatolia and Eastern Anatolia Regions. It is more prevalent in eastern part (3–4 animals per 100 km^2^) of Turkey than the west regions (1–2 animals per 100 km^2^) (Ambarli et al., 2016). The estimated number of *Canis lupus* is between 5000 and 7000 in Turkey (Can, 2001). There are some morphological and molecular studies for the detection of helminth species in gray wolves but no *Echinococcus* spp. have been recorded in gray wolves in Turkey, yet (Erol et al., 2021). However, there are many reports from other countries. 119 wolf carcasses were examined and checked for the occurrence of *E. granulosus* in Italy and 18 wolves resulted positive for *E. granulosus* (15%) (Guberti et al., 2004). Gray wolves (n = 123) were examined for *E. granulosus* in two states of USA. The worms were determined in 39 out of 63 animals (62%) in Idaho, and 38 out of 60 (63%) in Montana (Foreyt et al., 2009). The intestines of 27 Iberian wolves were investigated for the existence of *E. granulosus* and four animals (15%) were reported to be positive in Spain (Sobrino et al., 2006). In Latvia, the 34 hunted wolves were inspected, and *E. multilocularis* was determined in 5.9% (Bagrade et al., 2009). In a study on 26 wolf carcasses in Estonia, the NAD1 gene region was amplified by PCR and *E. granulosus* was detected in a sample (Moks et al., 2006). *Echinococcus canadensis* and *E. multilocularis* in Canada were identified on the basis of NAD1 gene sequence analysis from 30 wolves, and reported both *E. canadensis* (G8/10) and *E. multilocularis* in all sample collected areas (Schurer et al., 2014). In Portugal, stool samples were collected from 68 wolves and *E. granulosus* (G7) (1.5%) were detected by using of PCR and sequencing (Guerra et al., 2013). In another report, the intestines of 13 wolves were examined for the *Echinococcus* spp. in Poland. The mt-NAD1 and CO1 gene regions of the collected worm's were amplified and then sequenced. Finally, *E. ortleppi* (G5) was first time reported in a wolf in Poland (Karamon et al., 2021). In Mongolia, 118 wolves were examined and *E. multilocularis* was found in four wolves, whereas *E. canadensis* (G6/7) was defined in two and *E. canadensis* (G6/7) in three wolves (Ito et al., 2013). Intestines of 93 wolves in Canada were examined, and upon sequencing a 370 bp region of the mt-NAD1 gene, 17 new haplotypes were identified for *E. multilocularis* (Gesy et al., 2014).

The goal of the current work was to molecularly characterize and define of haplotypes using mt-CO1 gene sequences of adult *Echinococcus* worms collected from a gray wolf's intestine and contribute to the insufficient data on *Echinococcus* spp. status in wild animals in Turkey.

## 2 Materials and methods

### 2.1 Sample

A gray wolf (*Canis lupus*) was found dead by villagers at the rivers's edge in a rural area of Bingol province of Turkey and was brought to Veterinary Faculty of Bingol University. No precise data on the cause of death could be found. Their intestines were taken for parasitic examination and the remaining cadavers were received by the Anatomy Department for use in student practice. After a median insicion to abdomen the intestines were collected and waited at −80 °C for during two weeks to eliminate the dangerous contamination risk by inactivating any *Echinococcus* spp. eggs and then stored at −20 °C till to other analyses (Deplazes and Eckert, 1996). The small intestine was opened longitudinally and examined for visible helminths. The intestinal contents were washed, the mucosa was carefully scraped, and after successive decantation, helminths were searched under the stereoscope. Small cestodes (or cestode fragments) identified using a microscope (Soulsby, 1987). The Sedimentation Counting Technique (SCT) was used as described by Hofer et al. (2000). Briefly, the small intestine was incised longitudinally and cut into five pieces of roughly equal length. These pieces were placed in a glass bottle containing a 0.9% NaCl solution. After vigorously shaking the bottle for a few seconds, the intestine was removed and the superficial mucosal layer was stripped. After 15 min of sedimentation, the supernatant was decanted and the bottle was refilled with physiological saline solution. This was repeated 2–6 times until the supernatant was clear. The sediment fraction was examined under a stereomicroscope (Olympus, SZ51) in small portions of about 5–10 ml in petri dishes. Then, all parasites were counted, collected and stored in 70% ethanol for molecular identification. The intensity of infection classified as low (1–100), medium (101–1000), or high (1000<) worm burden according to Balog et al. (2021).

### 2.2 Genomic DNA isolation from the adult parasites

The gDNA's were isolated from the individual adult worms using a commercial kit (Hibrigen, Turkey) as recommended in the kit protocol, with a few minor changes. In brief, adult worms were putted into the eppendorf tubes (1.5-mL) and washed by 1X PBS having pH of 7.4 for at least six times to eliminate the excessive alcohol. Following, 200 μL of DL buffer, 15 μL (15 mg/mL) of Proteinase-K, and the individual worms were combined and incubated in waterbath (65 °C) during the night. The gDNA's that was isolated by kit protocol and stocked at −20 °C till PCR step.

### 2.3 PCR and sequence of the mt-CO1

The gDNA's of the adult tapeworms were molecularly analyzed using specific primers by PCR. The following primers F/CO1 (5′-TTGAATTTGCCACGTTTGAATGC-3′) and R/CO1 (5′-GAACCTAACGACATAACATAATGA -3′) were used for the amplifying of a part of mt-CO1 gene, that has been already reported by Nakao et al. (2000). The PCR was performed in a 50 μL reaction mix, which contained 5 μL 10X PCR buffer, 5 μL 25 mM MgCl_2_, 400 μM of each dNTP's, 20 pmol of each primers, 0.2 μL Taq polymerase enzyme (1.25 IU) (Hibrigen, Turkey), 28.8 μL PCR-grade water and 5 μL of the template gDNA. The PCR amplification protocol was implemented with some minor modifications reported by Kesik et al. (2021). The reaction was performed by using a thermal cycler (Blue-Ray Biotech Corp., Taiwan), and the products were separated using an agarose gel (1.5%) electrophoresis. The gel were visualised with RedSafe (iNtRON Biotech, South Korea) and photographed using ChemiDoc Imagers (Bio-rad, USA). After purifying of the PCR products, an unidirectionally sequence analysis was done using of the sense primer set (BM Labosis, Ankara, Turkey).

### 2.4 Alignment and phylogenetic analysis

A FinchTV 1.4.0 software (Geospiza Inc., Seattle Washington, USA) was used for the analyzing of the sequence results. Then a BLAST search were implemented to the sequence results using *E. granulosus* genome database at the NCBI. Following, the sequence data were trimmed then multiple sequence alignment and phylogenetic tree were established with MEGA X (Kumar et al., 2018). Clustal W tool was used for alignment of nucleotide sequences (Clustal et al., 1994). Previously published sequences of *E. granulosus s.l.* were used as the reference besides *Taenia saginata* and *E. multilocularis* were used as an outgroup sequences. Distance-based analyses were carried out via the Tamura-Nei-Gamma distribution (TN93 + G) model distance estimates, and trees were builded by Maximum Likelihood algorithm. Bootstrap analyses were performed using 1000 replicates (Kumar et al., 2018).

### 2.5 Haplotype networks, nucleotide polymorphism, diversity and neutrality indices

DnaSP6 software was performed for the additional analyses of the data (Rozas et al., 2017). Haplotype numbers (h), nucleotide diversity (π), and haplotype diversity (Hd) were detrmined as population diversity indices. Furthermore, impartiality indices (Tajima's D statistics, Fu's statistics), as well as Fu and Li's D and F values. The network was created using the PopART-1.7 software and the Minimum Spanning Networks (MSN) method (Leigh and Bryant, 2015).

## 3 Results

**Fig. 1:**
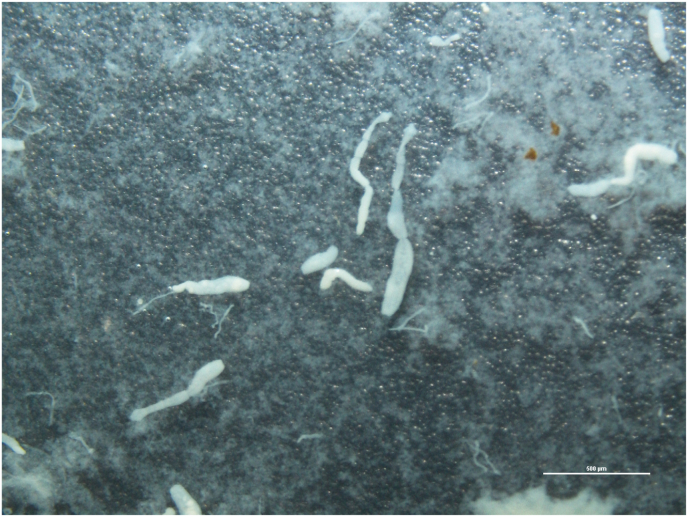
Stereomicroscopic view of adult parasites obtained from gray wolf's intestine.

Gray wolf (*Canis lupus*) had many adult *Echinococcus* spp. in its small intestine. However, most of them were destroyed because the carcass had been in the wild for a long time. A total of 78 scoleces were counted and many other parasite proglottids were seen. Therefore, only nine whole adult parasites with preserved parasite integrity were obtained. Morphological descriptions of the parasites were made under a stereo microscope (Fig. 1). Subsequently, gDNAs of all collected adult worms were analyzed by PCR and exact identifications of species were made as a result of the sequence analysis of a fragment of the mt-CO1 (875 bp).

### 3.1 Phylogenetic analysis

**Fig. 2:**
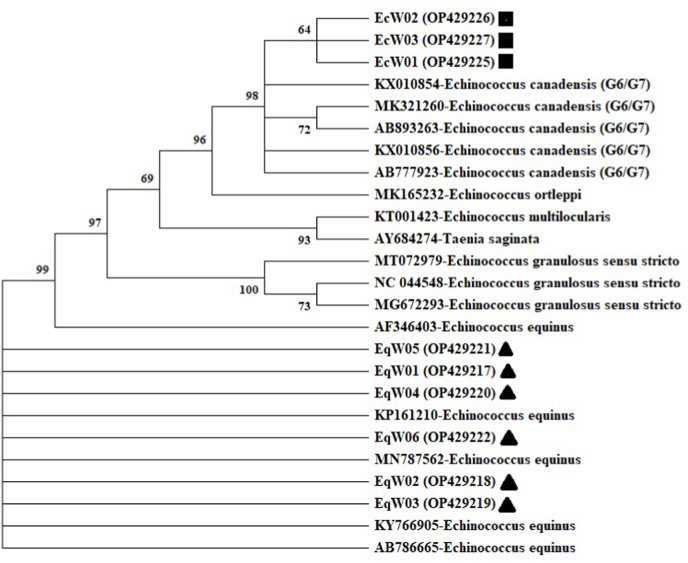
Phylogenetic tree of *Echinococcus granulosus s.l.* isolates generated using mt-CO1 gene sequences (815 bp). The phylogenetic tree was constructed using the Maximum Likelihood method and TN93 + G model. Evolutionary analyses were conducted in MEGA X. For each reference sequence, the GenBank accession number and species name are listed below: MN787562 (E. equinus), KY766905 (E. equinus), KP161210 (E. equinus) AB786665 (E. equinus) AF346403 (E. equinus), KX010854 (E. canadensis) MK321260 (E. canadensis) KX010856 (E. canadensis), AB893263 (E. canadensis), AB777923 (E. canadensis), MK165232 (E. ortleppi), MT072979 (E. granulosus s.s.), NC_044548 (E. granulosus s.s.), MG672293 (E. granulosus s.s.), KT001423 (E. multilocularis), AY684274 (T. sa*ginata*). ■: *E. canadensis* (G6/7) isolates; ▲: *E. equinus* isolates.

After the BLAST search, six sequences (Sample numbers: EqW01- EqW06) were characterized as *E. equinus*, while three sequences (Sample numbers: EcW01- EcW03) were identified as *E. canadensis* (G6/7). The sequences were then registered in GenBank database. The accession numbers were OP429217-OP429222 for *E. equinus* (EqW01-EqW06) and OP429225-OP429227 for *E. canadensis* (G6/7) (EcW01-EcW03). The genetic tree was created by aligning of the sequences that showed the phylogenetic relation with the representative sequences and out-groups were found through a BLAST search (Fig. 2)

### 3.2 Haplotype networks for E. equinus

Only three haplotypes were found in the haplotype network constructed using *E. equinus* mt-CO1 gene sequences from Turkey (OP429217– OP429222) in addition to *E. equinus* nucleotide sequences from Kyrgyzstan (dog, n = 1), Turkey (donkey, n = 1), Namibia (donkey, n = 4), UK (donkey, n = 1) and Australia (horse, n = 1) (Table 1). Fourteen sequences were convenient for haplotype network analysis, which presented three haplotypes (Hap_1-Hap_3) and Hap_1 dominated with 12 sequences (85%), and the resulting gray wolf isolates were included in this main haplotype. Hap_1 included isolates from Namibia, UK, Kyrgyzstan, and Turkey, while haplotype Hap_2 and Hap_3 included isolates from Namibia and Australia, respectively. Two haplotypes (Hap_2, Hap_3) were represented by only one sequence.

**Table 1:** Haplotypes of mt-CO1 sequences of *E. equinus* and accession numbers of isolates forming groups.

Haplotype Name	No. of Isolate	Accession Numbers (Origin, Host)
Hap_1	12	EqW01(OP429217) EqW02(OP429218) EqW03(OP429219) EqW04(OP429220) EqW05(OP429221) EqW06(OP429222) MN787562 (Kyrgyzstan, Dog) KY766905 (Turkey, Donkey) KP161210 (Namibia, Donkey) KP161208 (Namibia, Donkey) KP161207 (Namibia, Donkey) AB786665 (United Kingdom, Donkey)
Hap_2	1	KP161209 (Namibia, Donkey)
Hap_3	1	AF346403 (Australia, Horse)

**Fig. 3:**
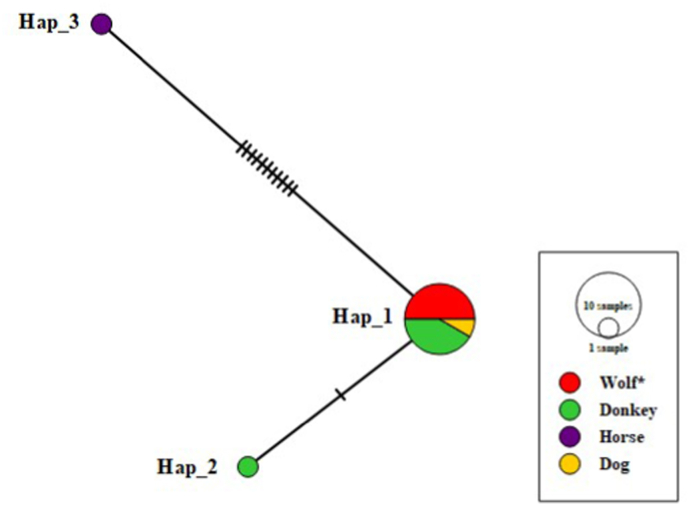
The haplotype network for the mt-CO1 gene (815 bp) of *E. equinus*. The size of the circles is proportional to the frequency of each haplotype. The number of mutations separating haplotypes is indicated by dash marks. The host diversity of haplotypes is shown in different colors. Hap: Haplotype.

One gene sequence reported from a horse in Australian (AF346403), which different by an eleven-step mutation from the *E. equinus* main haplotype (Hap_1), resided in the second *E. equinus* haplotype (Hap_3). The third *E. equinus* haplotype (Hap_2) was occupied by a sequence derived from an Namibia donkey isolate (KP161209) that diverged from the main *E. equinus* haplotype (Hap 1) by a one-step mutation. The haplotype network is shown in Fig. 3.

### 3.3 Nucleotide sequence variation, diversity and neutrality indices for E. equinus

The sequences contained 12 polymorphic sites that were not parsimony-informative. One conserved fragment (61–354) was detected in all of the sequenced isolates. The mean nucleotide variations were 0,00210 ± 0,00160, and haplotype diversities were 0,275 ± 0,148 for the mt-CO1 sequences. Tajima's D, which indicates population expansion and/or selection purification, was remarkably negative. The positive Fu's Fs value calculated for *E. equinus* community indicates haplotype/allele insufficiencies expected from recent population bottleneck events (Table 2).

**Table 2:** Diversity and neutrality indices obtained by using nucleotide data of mt-CO1 gene of *E. equinus and E. canadensis*.

Species	n	H	hd±SD	πd ± SD	Tajima'sD	P value	Fu's Fs	P value	FLD	P value	FLF	P value
*E. equinus*	14	3	0,275 ± 0148	0,00210 ± 0,00160	−2,17325	P < 0.01	2145	0,204	−2,85582	P < 0.02	−3,05921	P < 0.02
E. canadensis	119	47	0,880 ± 0026	0,00715 ± 0,00117	−1,97262	P < 0.05	−27,296	0,000	−2,01714	0.10 > P > 0.05	−2,39853	P < 0.05

n: Number of isolates, hn: number of haplotypes; hd: haplotype diversity; πd: nucleotide diversity; SD: standard deviation; FLD: Fu and Li's D test statistic.

FLF: Fu and Li's F test statistic.

### 3.4 Haplotype networks for E. canadensis (G6/7)

**Fig. 4:**
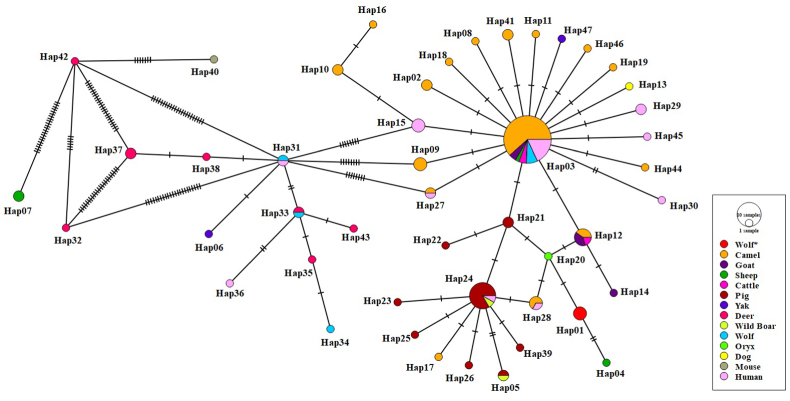
The haplotype network for the mt-CO1 gene (815 bp) of *E. canadensis* (G6/7). The size of the circles is proportional to the frequency of each haplotype. The number of mutations separating haplotypes is indicated by dash marks. The host diversity of haplotypes is shown in different colors. Hap: Haplotype.

A total of 47 haplotypes (Hap_01-Hap_47) discreted by one to twenty-six stepwise mutations were determined within the 119 *E. canadensis* (G6/7) mt-CO1 DNA sequences from Turkey and different host isolates in other geographic regions (Fig. 4) (Table 3). The network showed a star-like representation with a centrally positioned haplotype (Hap_03) constituting 32.7% (39/119) of the total number of isolates. Hap_01 consisted of the gray wolf isolates used in this study and was separated from the main haplotype (Hap_03) by three stepwise mutations. A BLAST search showed the *E. canadensis* (G6/7) Hap_01 to be 99.8% identical to the Hap_04 from the sheep isolate in Turkey (G6/G7; MT380299), 99.8% identical to the Hap_20 from the sheep isolate in Namibia (G6/G7; KX010854). The haplotype network is shown in Fig. 4.

**Table 3:** Haplotypes of mt-CO1 sequences of *E. canadensis* and accession numbers of isolates forming groups.

Haplotype Name	No. of Isolate	Accession Numbers (Origin, Host)
Hap01	3	EcW01 (OP429225) EcW02(OP429226) EcW03 (OP429227)
Hap02	2	NC011121 (Kazakhstan, Camel) AB208063 (Kazakhstan, Camel)
Hap03	39	AB688142 (Russia, Human) KR337822 (Iran, Goat)
		KX010830 (Kenya, Camel) KX010831 (Kenya, Camel)
		KX010835 (Kenya, Camel) KX010837(Kenya, Camel)
		KX010839 (Kenya, Camel) KX010848 (Sudan, Camel)
		KX010849 (Sudan, Camel) KX010850 (Sudan, Camel)
		KX010852 (Sudan, Camel) KX010871 (Kenya, Goat)
		MK321257 (Mongolia, Camel) MK321258 (Mongolia, Camel)
		AB893253 (Mongolia, Human) AB893255 (Mongolia, Human)
		AB893256 (Mongolia, Human) AB893257 (Mongolia, Human)
		AB893259 (Mongolia, Human) AB777909 (Russia, Wolf)
		AB777922 (Ethiopia, Camel) KP751428 (Iran, Camel)
		KP751429 (Iran, Camel) KP751430 (Iran, Camel)
		LC184603 (Mongolia, Camel) KX893477 (Iran, Camel)
		KX893479 (Iran, Human) MT166284 (Nigeria, Cattle)
		MT166285 (Nigeria, Camel) MT166286 (Nigeria, Camel)
		MT166288 (Nigeria, Camel) MT166289 (Nigeria, Camel)
		MT166290 (Nigeria, Camel) MN340039 (China, Sheep)
		MN025261 (Nigeria, Camel) MN025263 (Nigeria, Camel)
		MN025264 (Nigeria, Cattle) AB813182 (Mongolia, Wolf)
		AB813183 (Mongolia, Wolf)
Hap04	1	MT380299 (Turkey, Sheep)
Hap05	2	KX231667 (Armenia, Pig) KX010866 (Serbia, Wild Boar)
Hap06	1	MG597240 (China, Yak)
Hap07	2	MH791328 (China, Sheep) MH828449 (China, Sheep)
Hap08	1	KX010832 (Kenya, Camel)
Hap09	3	KX010833 (Kenya, Camel) MT166287 (Nigeria, Camel) MN025262 (Nigeria, Camel)
Hap10	2	KX010834 (Kenya, Camel) KX010840 (Kenya, Camel)
Hap11	1	KX010836 (Kenya, Camel)
Hap12	5	KX010838 (Kenya, Goat) KX010841 (Kenya, Goat)
		KX010851 (Sudan, Camel) KX010870 (Ethiopia, Cattle) AB777923 (Ethiopia, Camel)
Hap13	1	KX010842 (Kenya, Dog)
Hap14	1	KX010843 (Kenya, Goat)
Hap15	3	KX010844 (Sudan, Human) AB893254 (Mongolia, Human) AB893260 (Mongolia, Human)
Hap16	1	KX010845 (Sudan, Camel)
Hap17	1	KX010846 (Sudan, Camel)
Hap18	1	KX010847 (Sudan, Camel)
Hap19	1	KX010853 (Sudan, Camel)
Hap20	1	KX010854 (Namibia, Oryx)
Hap21	2	KX010855 (France, Pig) KX010856 (France, Pig)
Hap22	1	KX010857 (France, Pig)
Hap23	1	KX010858 (Slovakia, Pig)
Hap24	12	KX010859 (Slovakia, Pig) KX010860 (Hungary, Pig)
		KX010861 (Hungary, Pig) KX010862 (Romania, Wild Boar)
		KX010863 (Serbia, Pig) KX010864 (Serbia, Pig)
		KX010868 (Serbia, Pig) KX010869 (Kenya, Human)
		AB777924 (Peru, Pig) AB777925 (Peru, Pig)
		KX510133 (Serbia, Pig) KX510134 (Serbia, Pig)
Hap25	1	KX010865 (Serbia, Pig)
Hap26	1	KX010867(Serbia, Pig)
Hap27	2	KX010872 (Kenya, Camel) AB893252 (Mongolia, Human)
Hap28	3	MK321259 (Mongolia, Camel) MK321260 (Mongolia, Camel)
		AB893263 (Mongolia, Human)
Hap29	2	AB893258 (Mongolia, Human) AB893262 (Mongolia, Human)
Hap30	1	AB893261 (Mongolia, Human)
Hap31	2	AB893264 (Mongolia, Human) AB813184 (Mongolia, Wolf)
Hap32	1	AB777910 (Russia, Deer)
Hap33	2	AB777911 (Russia, Deer) AB813185 (Mongolia, Wolf)
Hap34	1	AB777912 (Russia, Wolf)
Hap35	1	AB777913 (Russia, Deer)
Hap36	1	AB777914 (Russia, Human)
Hap37	2	AB777926 (USA, Deer) LC184606 (USA, Deer)
Hap38	1	AB777927 (USA, Deer)
Hap39	1	AB235847 (Japan, Pig)
Hap40	1	AB235848 (Japan, Mouse)
Hap41	2	KP751426 (Iran, Camel) KP751427 (Iran, Camel)
Hap42	1	LC184604 (Russia, Deer)
Hap43	1	LC184605 (Russia, Deer)
Hap44	1	KX893476 (Iran, Camel)
Hap45	1	KX893478 (Iran, Human)
Hap46	1	KX893480 (Iran, Camel)
Hap47	1	MN340038 (China, Yak)

### 3.5 Nucleotide sequence variation, diversity and neutrality indices for E. canadensis

The mt-CO1 sequences contained 76 polymorphic regions, of which 67% (51/76) were parsimony-informative. Five conserved fragments (298–332, 461–500, 559–593, 671–713 and 770–773 bp) were detected in all sequences. The overall base changes were 0,00715 ± 0,00117, and haplotype variation were 0,880 ± 0,026 for the mt-CO1 sequences. Tajima's D negativity was important for *E. canadensis* populations, indicating the presence of variant nucleotides and population expansion (Tajima's D −1,97262, p < 0.05). This was also supported by the negative Fu's Fs value (−27,296) for *E. canadensis* populations, indicating the occurrence of extra alleles as anticipated following recent population expansion or genetic hitchiking (Table 2).

## 4 Discussion

This study is important as it is the first study in which *E. equinus* and *E. canadensis* (G6/7) were found together as a result of genotyping of adult *Echinococcus* species obtained from a gray wolf in Turkey. Some *Echinococcus* species have life cycles involving domestic animals, while others have wildlife cycles that may or may not interact with domestic contamination. Wildlife contamination may originate from domestic animals or directly in the wildlife cycle (Romig et al., 2017). The available information in Turkey does not yet allow for a precise definition of transmission systems of *E. granulosus s.s*. It especially well adapted to sheep, but the ancient wildlife cycle may have contained wild animals (wild sheep and goats, cervids, and wild boars) as intermediate hosts besides wolves and golden jackals as final hosts. However, given that few wild host species remain, these infection records are likely to indicate spillover events from this region's ubiquitous domestic life cycle.

Previous studies have shown that *E. equinus* has a special sylvatic cycle (Wassermann et al., 2015). It has been reported from Namibia that lions and black-backed jackals act as final hosts, and lowland zebras are metacestode bearer. It is estimated that this species finds its distribution area by taking advantage of the predatory prey system between zebras and lions, and coyotes get the infection by eating carrion (Wassermann et al., 2015). However, until today, no data has been found regarding the presence of *E. equinus* in wolves.

Epidemiological studies done in distinct localities of the world to determine the host role of wolves, but they are quite limited. The existence of *E. granulosus* in Kazakhstan, *E. intermedius* (G7) in Portugal, *E. granulosus* and *E. multilocularis* in Italy, and *E. canadensis*/*E. multilocularis* mix infections in wolves have been recorded in Canada and Mongolia (Guberti et al., 2004; Abdybekova and Torgerson, 2012; Guerra et al., 2013; Ito et al., 2013; Schurer et al., 2014; Massolo et al., 2018). Now, *E. granulosus s.s.*, *E. equinus, E. ortleppi*, *E. canadensis* (G6/G7) and *E. multilocularis* circulate in human and animals in Turkey (Inceboz et al., 2005; Avcioglu et al., 2016, 2021; Kesik et al., 2020). *E. canadensis* (G6/7) has been reported in sheep, cattle, and humans (Mehmood et al., 2020; Bakal et al., 2021; Kesik et al., 2021), while *E. equinus* has been found in donkeys, mules and humans in some previous studies in Turkey (Simsek and Cevik, 2014; Kesik et al., 2019; Macin et al., 2021). In the current study, *E. canadensis/E.equinus* co-infections were first time detected in a gray wolf in Turkey. Of the 9 isolates obtained from the gray wolf, six isolates were determined as *E. equinus* and three isolates as *E. canadensis*. The sequence analysis of two *Echinococcus* spp. (collected from a single gray wolf) revealed distinct genotypes at the mt-CO1 gene, indicating that the gray wolf most probably acquired the worms from multiple sources.

The isolate of *E. equinus* obtained from the gray wolf carried a mt-CO1 sequence 100% identical to the donkey isolate in Turkey (Kinkar et al., 2017). It also showed identical sequence to the donkey isolate from Namibia and the dog isolate from Kyrgyzstan (Wassermann et al., 2015; Alvarez Rojas et al., 2020). Only three haplotypes were found in the haplotype network constructed using *E. equinus* mt-CO1 gen sequences. Hap_1 was found in isolates from different geographical regions. It was quite closely related to the genotype previously identified from Namibia, United Kingdom, Kyrgyzstan and Turkey, suggesting that the haplotype could be termed Palearctic and Afrotropic genotype. Hap_2 and Hap_3 were same to more sequences from Namibia and Australia, respectively. This wide distribution is consistent with a high probability of transmission by final hosts; for instance, wolves can cover more than 62,000 km^2^ (Walton et al., 2001). This idea is supported by clustering some closely related isolates collected in close surroundings. In addition, the anthropogenic translocation of wolves for hunting may also support to the spread of the worms in some regions (Foreyt et al., 2009).

A total of 47 haplotypes were found in the haplotype network constructed using *E. canadensis* mt-CO1 sequences from Turkey and other regions. The strain of *E. canadensis* (G6/7) from the wolf in Turkey carried out a mt-CO1 sequence 99.8% identical to *E. canadensis* (G6/7) identified from sheep in Turkey (Mehmood et al., 2020). Hap_01 was separated from Hap_04 by two point mutations. Three isolates obtained from a wolf separated from all other haplotypes and formed a different haplotype. The haplotype furthest from hap_01 was *E. canadensis* (G8) in Russia (Konyaev et al., 2013; Yanagida et al., 2017).

The G6 genotype of *E. canadensis* has traditionally been considered as 'camel strain'. There are 1315 camels, and very few camels bred in Turkey are used as pack animals (Yılmaz et al., 2014). Thus, the G6 genotype is unlikely to be provided only by a wild gray wolf-camel life cycle. Considering that cysts of *E. canadensis* (G6/7) have been found in livestock animals in Turkey, it is conceivable that the gray wolf may obtaine the *E. canadensis* (G6/7) worms by consuming these animal's infected organs.

## 5 Conclusions

This study defines the first report of an *E. equinus/E. canadensis* co-infection in a gray wolf. It is currently unknown whether these wolves are entirely wild, becoming infected by prey species, or if they are spilling over from the domestic cycle via predation of livestocks or scavenging carcasses. This study will pave the way for future research on both the definitive host of wolves and the prevalence of *Echinococcus* spp. in gray wolves in Turkey.

## Declaration of competing interest

All authors drafted the manuscript and revised it for final approval.
